# Ion-Beam Synthesis of Gallium Oxide Nanocrystals in a SiO_2_/Si Dielectric Matrix

**DOI:** 10.3390/nano12111840

**Published:** 2022-05-27

**Authors:** Dmitry S. Korolev, Kristina S. Matyunina, Alena A. Nikolskaya, Ruslan N. Kriukov, Alexey V. Nezhdanov, Alexey I. Belov, Alexey N. Mikhaylov, Artem A. Sushkov, Dmitry A. Pavlov, Pavel A. Yunin, Mikhail N. Drozdov, David I. Tetelbaum

**Affiliations:** 1 Research Institute of Physics and Technology, Lobachevsky State University of Nizhny Novgorod, 603022 Nizhny Novgorod, Russia; nikolskaya@nifti.unn.ru (A.A.N.); belov@nifti.unn.ru (A.I.B.); mian@nifti.unn.ru (A.N.M.); tetelbaum@phys.unn.ru (D.I.T.); 2 Department of Physics, Lobachevsky State University of Nizhny Novgorod, 603022 Nizhny Novgorod, Russia; matyunina.ks@gmail.com (K.S.M.); kriukov.ruslan@yandex.ru (R.N.K.); nezhdanov@phys.unn.ru (A.V.N.); sushkov@phys.unn.ru (A.A.S.); pavlov@unn.ru (D.A.P.); yunin@ipmras.ru (P.A.Y.); 3 Institute for Physics of Microstructure of the Russian Academy of Sciences (IPM RAS), 603950 Nizhny Novgorod, Russia; drm@ipm.sci-nnov.ru

**Keywords:** gallium oxide, nanocrystals, silicon oxide, ion-beam synthesis, ion implantation, thermal annealing, photoluminescence

## Abstract

A new method for creating nanomaterials based on gallium oxide by ion-beam synthesis of nanocrystals of this compound in a SiO_2_/Si dielectric matrix has been proposed. The influence of the order of irradiation with ions of phase-forming elements (gallium and oxygen) on the chemical composition of implanted layers is reported. The separation of gallium profiles in the elemental and oxidized states is shown, even in the absence of post-implantation annealing. As a result of annealing, blue photoluminescence, associated with the recombination of donor–acceptor pairs (DAP) in Ga_2_O_3_ nanocrystals, appears in the spectrum. The structural characterization by transmission electron microscopy confirms the formation of β-Ga_2_O_3_ nanocrystals. The obtained results open up the possibility of using nanocrystalline gallium oxide inclusions in traditional CMOS technology.

## 1. Introduction

Wide-gap semiconductor materials are currently attracting attention from the viewpoint of developing new-generation electronic devices, the creation of which is difficult or impossible using traditional silicon technology. One of the most promising wide-gap materials is gallium oxide, which, due to its unique properties, such as a large band gap (~4.5–5 eV), can serve as the basic material for power electronics devices, solar-blind photodetectors, gas sensors, etc. [1,2,3,4]. An important feature of this material is the presence of several polymorphic modifications, in particular, the monoclinic (β-Ga_2_O_3_), defective spinel (γ), rhombohedral (α), cubic (δ), or orthorhombic (ε). The monoclinic β-Ga_2_O_3_ phase is the most stable under normal conditions and attracts the most attention. However, other polymorphs are also of interest due to their different physical properties from β-Ga_2_O_3_ [5,6]. Despite its unique characteristics, the use of gallium oxide has not yet become widespread, due to the complexity of growth technology and the high cost of single-crystal substrates, as well as the insufficient maturity of technologies for obtaining thin films on crystalline substrates [7,8].

A separate niche among Ga_2_O_3_ technologies is preoccupied with the creation and study of nanostructured materials, e.g., nanosized Ga_2_O_3_ inclusions of various polymorphic modifications [9]. The creation of nanosized Ga_2_O_3_ inclusions significantly expands the possibilities of practical application of this material. For example, Ga_2_O_3_ nanocrystals (nc-Ga_2_O_3_) demonstrate high catalytic performance for heterogeneous catalysis [10,11] and the creation of low-cost UV-to-visible converters for monitoring UV-emitting events on a large-scale—from invisible hydrogen flames to corona dispersions [12] and solar cells [13], as well as when creating photonic devices [14]. Along with the abovementioned applications, special attention is given to the possibility of using gallium oxide nanocrystals in devices with luminescence in the UV and visible spectral regions.

A large number of studies are devoted to the synthesis of nanopowders and colloidal solutions of nc-Ga_2_O_3_ using chemical methods [15,16,17]. It has been shown that the emission associated with recombination at structural defects is predominant in their luminescence spectrum [12]. The effect of synthesis conditions on the size of nanocrystals has been studied, and the dependence of the position of luminescence peaks on the size of nanoinclusions was found [18]. The efficiency of using nanocrystals of the wide-gap Ga_2_O_3_ semiconductor as a matrix for embedding rare-earth ions and obtaining the corresponding luminescence lines has also been demonstrated [19,20].

Despite all the advantages of chemical synthesis methods, their use in the fabrication of devices using traditional CMOS technology is practically impossible. In this work, we propose a new method for creating nc-Ga_2_O_3_ by ion-beam synthesis of nanoinclusions in solid-state matrices. This method is fully compatible with CMOS technology and has the ability to controllably change the properties of created structures by varying the parameters of ion synthesis. The ion implantation method has already demonstrated its effectiveness in β-Ga_2_O_3_ technology [21]. However, in [21], attention was mainly paid to the overview of “traditional” technological applications of ion implantation, including the issues of radiation defect formation during the irradiation of gallium oxide and the prospects of ion implantation for controlled doping of Ga_2_O_3_ single crystals and thin-film structures. In the framework of this manuscript, another aspect of the application of this technology is considered, related to the ion-beam synthesis of gallium oxide nanocrystals in solid-state dielectric matrices.

Ion-synthesized nanoinclusions of semiconductors such as Si [22], Sn [23], ZnO [24], In_2_O_3_ [25] and many others have been previously reported. However, the ion synthesis of nc-Ga_2_O_3_ was practically not reported, except in the case of our work, which demonstrated the possibility of synthesizing such nanocrystals in an Al_2_O_3_ matrix [26]. At the same time, questions about the influence of the matrix and parameters of ion-beam synthesis on the structure and composition of nc-Ga_2_O_3_ remained unexplored, and the light-emitting properties of such structures have not been studied.

This article presents the results of a comprehensive study of the processes of ion-beam synthesis of nc-Ga_2_O_3_ in a silicon oxide matrix on a silicon substrate (SiO_2_/Si) under varying regimes of ion implantation and subsequent annealing, as well as their structure and luminescent properties.

## 2. Materials and Methods

**Figure 1:**
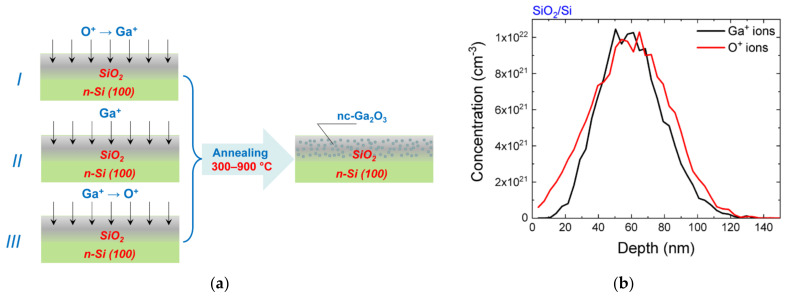
(**a**) Scheme of used variants of ion-beam synthesis of nc-Ga_2_O_3_; (**b**) SRIM-calculated distribution of implanted gallium and oxygen ions.

SiO_2_ films deposited on *n*-Si (100) silicon wafers by electron-beam evaporation were used as initial samples. The film thickness was ~350 nm. Ion-beam synthesis included two successive stages: irradiation with ions of phase-forming elements and subsequent high-temperature annealing (Figure 1a). In the first stage of the synthesis, implantation of Ga^+^ (80 keV, 5 × 10^16^ cm^−2^) and O^2+^ (45 keV, 3 × 10^16^ cm^−2^) ions was carried out with variation in the irradiation order. Only gallium ion implantation was used, since the participation of oxygen from the oxide matrix in the formation of nc-Ga_2_O_3_ was expected. Irradiation regimes were selected based on the condition of proximity of ion distribution profiles calculated using SRIM code (www.srim.org) and ensured the maximum implanted atoms at a depth of ~60 nm (Figure 1b). The implantation of molecular oxygen ions instead of atomic ions was used to reduce the total irradiation time. When colliding with the sample surface, O_2_^+^ molecular ions decomposed to form two ions with half the energy. In the second stage of ion synthesis, sequential 30 min isochronal annealing at 300, 500, 700 and 900 °C in a tube furnace in dried N_2_ atmosphere was carried out.

The study of the composition and chemical state of atoms in the initial samples was carried out by X-ray photoelectron spectroscopy (XPS) using an ultrahigh-vacuum complex Omicron Multiprobe RM (Omicron, Germany). The photoelectron lines O 1*s*, C 1*s*, Ga 2*p*_3/2_ and Si 2*s* were registered. The data collection area was ~7 mm^2^. The spectra were recorded at an analyzer transmission energy of 30 eV and an energy discretization of 0.2 eV/step. To obtain the depth distribution profiles of chemical elements, etching with Ar^+^ ions with energy of 1 keV and an etching region diameter of 20 mm was used. The ion beam axis was an angle of 45° to the normal of the samples.

The Raman scattering spectra were studied in the reflection scheme in the range of 50–900 cm^–1^ with a resolution of 0.7 cm^–1^. All spectra were measured at room temperature. The spectra were detected using a cooled CCD array (data are shown in Figure S1 of Supplementary Materials).

X-ray diffraction measurements were made on a BRUKER D8 Discover diffractometer (Cu Kα radiation). A LynxEye Linear PSD detector was used.

The study of photoluminescence spectra was carried out according to the standard lock-in technique. Plasma light source XWS-65 (TRDC, Moscow, Russia) with a filter that selects a region ~5 nm wide from the spectrum with the maximum at a wavelength of 245 nm was used as an excitation source. The spectra were recorded at room temperature.

The depth distribution of impurities was studied by time-of-flight secondary ion mass spectrometry (SIMS) with layer-by-layer analysis on a ToF.SIMS-5 spectrometer (IONTOF GmbH, Münster, Germany). Further, 25 keV Bi^3+^ ions were used as a probing beam, and 1 keV Cs^+^ ions were used as a profiling beam.

The structure of the samples was studied by cross-sectional transmission electron microscopy on a JEOL JEM-2100F microscope using a Gatan 601.07000 TEM Specimen Preparation Kit for sample preparation according to the Gatan method (Pleasanton, CA, USA).

## 3. Results and Discussion

### 3.1. Composition of As-Implanted Samples

**Figure 2:**
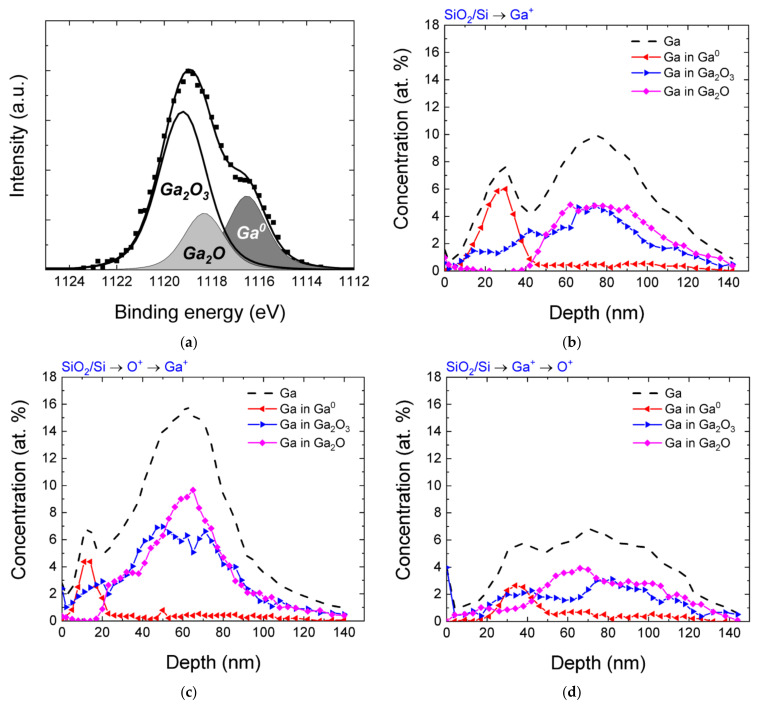
(**a**) An example of the decomposition of the Ga 2*p_3/2_* photoelectron line; (**b**–**d**) depth distribution profile of gallium (dashed line), as well as gallium in different chemical states (Ga^0^, Ga_2_O_3_, Ga_2_O, colored lines) for different implantation orders.

The distribution profiles of implanted gallium atoms without annealing obtained by the XPS method are shown in Figure 2. The figure also shows the distribution profiles of chemical bonds obtained by analyzing photoelectron lines with allowance for chemical shifts. An example of the decomposition of the Ga 2*p*_3/2_ photoelectron line is also shown in Figure 2. In addition to the presence of bonds corresponding to gallium in the elemental state, the formation of oxidized gallium in the states of the stoichiometric oxide Ga_2_O_3_ and in the state with a lack of oxygen Ga_2_O is also observed in the samples. It is clearly observed that the gallium profile has two maxima. The first one mainly contains gallium in the elemental state and is closer to the surface. The second maximum is located at depths close to the maximum of the implanted impurity distribution and deeper, while almost all of the implanted gallium at these depths is in the oxidized state. As can be observed from Figure 2, the distribution of gallium atoms in the sample significantly depends on the order of implantation. Thus, in a sample implanted with gallium only, the highest content of Ga-Ga bonds is observed, but even in the absence of additional oxygen irradiation, the sample also contains gallium in the oxidized state. This shows that the formation of Ga-O bonds actively consumes oxygen contained in the initial SiO_2_ matrix, and this occurs even in the absence of thermal annealing.

In the case of the implantation of O^+^ ions prior to implantation of Ga^+^, an increase in the total amount of gallium, a shift in the distribution profile towards shallower depths, and a closer coincidence of the position of the second (volume) profile maximum with the SRIM-calculated maximum (Figure 1) are observed. This may be due to the fact that preliminary irradiation with oxygen ions leads to the appearance of radiation defects in the implanted layer, which prevent radiation-accelerated diffusion of the implanted gallium. The presence of additionally implanted oxygen leads to an increase in the concentration of gallium in the oxidized state, and in the region of the maximum calculated distribution of implanted gallium, a sharp increase in the concentration of Ga-O bonds with a lack of oxygen is observed. The reason for this effect may be the limited oxygen content in this layer, which can be spent on the oxidation of gallium, whose concentration is at the maximum at these depths. Upon implantation of oxygen after implantation of Ga^+^ ions, the situation is generally similar to that observed upon implantation of gallium only; however, the concentration of Ga-Ga bonds is low, which indicates that additional incorporation of oxygen in the Ga-enriched layer leads to additional oxidation of gallium atoms.

### 3.2. Light-Emitting Properties of As-Implanted and Annealed Samples

**Figure 3:**
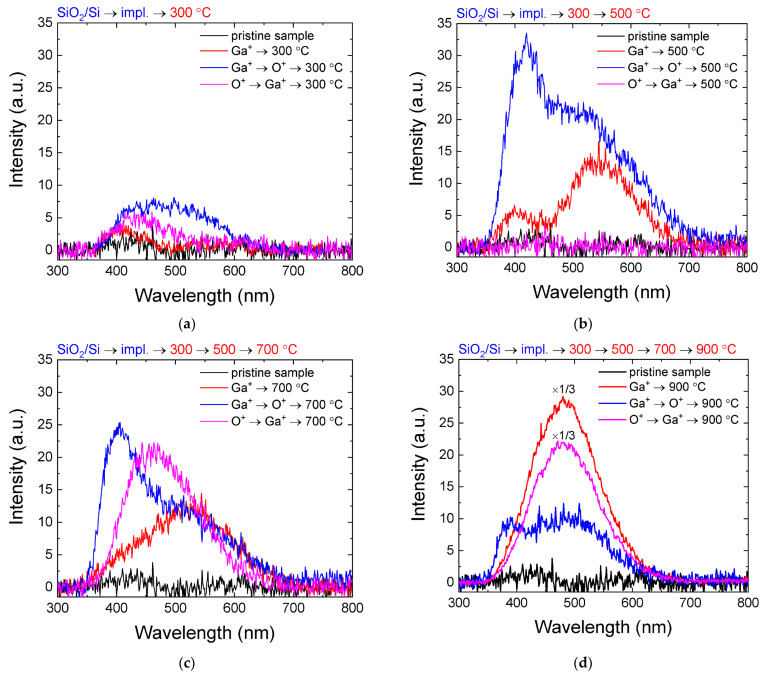
PL spectra of SiO_2_/Si samples irradiated with gallium and oxygen ions in different regimes after sequential annealing: (**a**) at 300 °C; (**b**) at 500 °C; (**c**) at 700 °C; (**d**) after final annealing at 900 °C.

Let us turn to the consideration of the light-emitting properties of the synthesized samples. To study the photoluminescence (PL) spectra, a source with a wavelength of ~245 nm (~5 eV) was used, which provides band-to-band optical excitation. The PL measurement of the implanted samples without annealing does not reveal the appearance of any luminescent lines. Annealing at 300 °C leads to the appearance of broad luminescent lines in the 400–550 nm region, and the broadest and most intense line is only observed for the sample with the Ga^+^ → O^+^ implantation order (Figure 3). An increase in the annealing temperature to 500 °C is accompanied by the appearance of lines with maxima at ~400 nm and ~500 nm, as well as an increase in the luminescence intensity and a shift in the long-wavelength maximum to ~520–530 nm. PL also appears in the sample irradiated with Ga ions only. The appearance of a short-wavelength maximum at a wavelength of ~400 nm can be due to both luminescence in gallium oxide, associated with exciton recombination [12], and defect-related luminescence in a silicon oxide matrix damaged by irradiation [27]. Investigation of the nature of this line requires separate studies; therefore, we will only consider the long-wavelength peak. The next step of annealing at 700 °C leads to a blue shift in the maximum, as well as the appearance of intense PL for the sample, which was irradiated with oxygen before Ga^+^ implantation. In this case, the maximum of the PL line shifts towards short wavelengths, relative to other samples. For some types of structures, the luminescence intensity increases significantly after the final annealing at 900 °C, which may indicate an increase in the concentration of the formed nanoclusters. The PL maximum shifts to the ~480 nm region, and its position turns out to be practically the same for all the studied samples.

Let us consider the possible reasons for the observed regularities. The long-wavelength maximum coincides with the position of the main Ga_2_O_3_ luminescent band, and is related to radiative recombination at donor and acceptor pairs (DAP) [12,28]. An oxygen vacancy (V_O_) acts as a donor, and a defect complex consisting of an oxygen and gallium vacancies pair (V_Ga_, V_O_) acts as an acceptor. The change in the position of the wavelength maximum is determined by the size of nanocrystals—an increase in the average size leads to a long-wavelength shift of the PL maximum [18].

In our case, we can assume the following mechanism for changing the luminescent properties with a change in the properties of the synthesized nanocrystals. As already confirmed above by the XPS method, the formation of Ga-O bonds in the samples occurs immediately after implantation. Annealing at a relatively low temperature (300 °C) is accompanied by the formation of non-phase inclusions or chains of Ga-O bonds, which practically do not affect the PL spectra. An increase in the annealing temperature to 500 °C leads to the growth of the clusters. A further increase in the annealing temperature practically does not change the PL intensity, but is accompanied by a slight shift in the maximum position to shorter wavelengths, which may indicate both the decomposition of large particles and the growth of smaller particles, or a possible phase transformation of nc-Ga_2_O_3_. It is interesting that the appearance of PL in a sample irradiated first with oxygen and then with gallium occurs only at this temperature, and the position of the maximum is significantly shifted to the left to a ~480 nm wavelength. The final annealing at 900 °C leads to the coincidence of the maximum position for all the studied samples with a simultaneous increase in the PL intensity, which may indicate an increase in the volume fraction of nc-Ga_2_O_3_ of the optimal size. Of course, the presented scheme requires additional study of the structural transformations that occur during annealing and accompany the evolution of nc-Ga_2_O_3_.

### 3.3. Direct Observation of nc-Ga_2_O_3_

**Figure 4:**
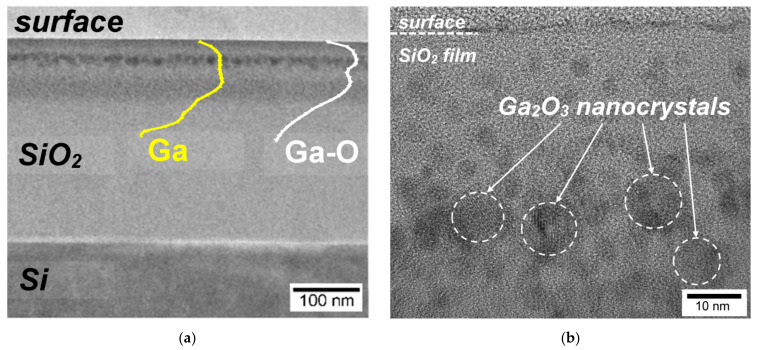
(**a**) Cross-sectional image of the SiO_2_/Si: (Ga^+^ + O^+^) structure after final annealing at 900 °C. The yellow and white curves show the distribution profiles of elemental and oxidized gallium, respectively, obtained by the SIMS method; (**b**) HRTEM image for the same sample on which synthesized nc-Ga_2_O_3_ was highlighted.

As direct evidence of the formation of nc-Ga_2_O_3_, the method of transmission electron microscopy (TEM), including high-resolution transmission electron microscopy (HRTEM), was used. Figure 4 shows the cross-sectional images of the SiO_2_/Si: (Ga^+^ + O^+^) structure after final annealing at a temperature of 900 °C. An observed TEM image (Figure 4a) shows the formation of two layers with dark contrast. In a narrow layer closer to the surface, the formation of large spherical nanoinclusions is observed, while at greater depths, the formation of a wider layer with small particles is observed. Comparison with the SIMS data (yellow and white curves in Figure 4a for the gallium lines in the atomic state and in the bond with oxygen, respectively) shows that these layers are enriched with gallium. The yield of gallium in the elemental state gives a more uniform distribution in the implanted layer and corresponds to the average distribution of gallium in all states, while the distribution of oxidized gallium corresponds to the bimodal distribution obtained by XPS.

The HRTEM image (Figure 4b) shows the formation of a large number of spherical nanoinclusions with dark contrast. For inclusions for which atomic planes are observed in the TEM patterns, the interplanar distances were measured. The obtained values were 2.82 and 2.41 A, which correspond to the (002) and (401) planes for the β-Ga_2_O_3_ phase (JCPDS Card No. 41-1103). Corresponding weak reflection spots for such planes are also observed in the electron diffraction image (Figure S2). This agrees well with the X-ray diffraction data, for which a weak peak at 2θ = 31.5° appears only after annealing at 900 °C (Figure S3). The peak position is consistent with the reflection for the (002) planes of β-Ga_2_O_3_. However, it can be observed from the TEM images that the volume fraction of nc-Ga_2_O_3_ is quite low, which explains the low signal intensities in the study of samples by Raman scattering and X-ray diffraction (see Supplementary Materials) [29,30,31].

## 4. Conclusions

The process of ion-beam synthesis of nc-Ga_2_O_3_ in the SiO_2_/Si dielectric matrix has been studied. It is shown that the formation of Ga-O bonds occurs even in the absence of post-implantation annealing, and their formation significantly depends on the order of ion irradiation. The study of the structural features of the samples after annealing suggests that the formation of nanoinclusions proceeds in several stages. First, the formation of non-phase inclusions of gallium oxide occurs, which, with an increase in the annealing temperature, are transformed into highly defective inclusions of the cubic γ-phase. With a further increase in temperature, the average size of the nanocrystals decreases, accompanied by a gradual transition of inclusions to the β-phase, which is almost completely finished after annealing at 900 °C. This assumption partly follows on from the photoluminescence data, for which a shift in the maximum is observed, apparently due to the size effect. The main mechanism of the observed nc-Ga_2_O_3_ luminescence is attributed to DAP recombination, where an oxygen vacancy acts as a donor, and a pair of gallium and oxygen vacancies acts as an acceptor.

Thus, the prospect of using the ion-beam synthesis technique is demonstrated to create nc-Ga_2_O_3_ with intense luminescence in the blue part of the spectrum. The possibility of controlling the properties of formed particles by varying the parameters of ion-beam synthesis is shown. An important advantage of the proposed technique is the complete compatibility of the used materials and the applied technological operations with traditional CMOS technology. This opens up the possibility of wider study and application of this technique to create a new generation of electronic and optical devices, using gallium oxide as a basic material.

## Data Availability

The data presented in this study are available on request from the corresponding authors.

## Supplementary Materials

The following supporting information can be downloaded at: https://www.mdpi.com/article/10.3390/nano12111840/s1, Figure S1. Raman scattering spectra for a SiO_2_/Si: (Ga^+^ + O^+^) sample, before and after sequential annealing. For comparison, the spectra of the initial Si substrate and the initial SiO_2_/Si film are also shown. Positions of the unidentified lines are indicated by arrows. Spectra are shown after removing the luminescent background. On the inset, the non-corrected spectra are shown. Figure S2. Electron diffraction picture of the SiO_2_/Si: (Ga^+^ + O^+^) structure after final annealing at 900 °C. Spots corresponding to (002) and (401) planes of the nc-Ga_2_O_3_ (β-phase) are indicated on the picture. Figure S3. X-ray diffraction spectra of the SiO_2_/Si samples implanted with Ga^+^ and O^+^ ions after final annealing at 900 °C.
