# Supplementary material for: Ion-Beam Synthesis of Gallium Oxide Nanocrystals in a SiO2/Si Dielectric Matrix
Source: Nanomaterials (Basel). 2022 May 27;12(11):1840. doi: 10.3390/nano12111840 (PMC9182438; doi:10.3390/nano12111840)
Supplement: Supplementary file 1 [file nanomaterials-12-01840-s001.zip › nanomaterials-1720642-supplementary.pdf]

Supplementary materials

Ion-Beam Synthesis of Gallium Oxide Nanocrystals in a SiO_2/Si Dielectric Matrix

Dmitry S. Korolev [1,*], Kristina S. Matyunina [2], Alena A. Nikolskaya [1,2], Ruslan N. Kriukov [2], Alexey V. Nezhdanov [2], Alexey I. Belov [1], Alexey N. Mikhaylov [1], Artem A. Sushkov [2], Dmitry A. Pavlov [2], Pavel A. Yunin [2,3], Mikhail N. Drozdov [3] and David I. Tetelbaum [1]

[1] Research Institute of Physics and Technology, Lobachevsky State University of Nizhny Novgorod, 603022 Nizhny Novgorod, Russia; nikolskaya@nifti.unn.ru (A.A.N.); belov@nifti.unn.ru (A.I.B.); mian@nifti.unn.ru (A.N.M.); tetelbaum@phys.unn.ru (D.I.T.)

[2] Department of Physics, Lobachevsky State University of Nizhny Novgorod, 603022 Nizhny Novgorod, Russia; matyunina.ks@gmail.com (K.S.M.); kriukov.ruslan@yandex.ru (R.N.K.); nezhdanov@phys.unn.ru (A.V.N.); sushkov@phys.unn.ru (A.A.S.); pavlov@unn.ru (D.A.P.); yunin@ipmras.ru (P.A.Y.)

[3] Institute for Physics of Microstructure of the Russian Academy of Sciences (IPM RAS), 603950 Nizhny Novgorod, Russia; drm@ipm.sci-nnov.ru

* Correspondence: dmkorolev@phys.unn.ru

Supplementary note 1. Raman scattering for as-implanted and annealed samples

The results of studying the samples by Raman scattering are shown in Fig. S1. All spectra were obtained under the same conditions (wavelength and excitation power, exposure time). For the original and irradiated substrates without annealing, peaks are observed from crystalline silicon only. The figure shows the phonon modes from the silicon substrate: second-order transverse acoustic (2TA) at ~300 cm^{-1}, transverse optical (TO) at ~520 cm^{-1}, and a wide area at 600–700 cm^{-1} from the second-order longitudinal acoustic (2LA) mode. All measured curves were scaled (normalized) by the intensity of the TO mode.

Upon annealing, the appearance of background caused by the appearance of photoluminescence in the samples is observed (inset to Fig. S1). However, against the background of the shoulder from photoluminescence, peaks are observed on the annealed samples, which are not characteristic of the lines from the silicon substrate. For a more detailed analysis, the luminescent background was removed (Fig. S1). It is known that, depending on the polytypes, gallium oxide has a different set of Raman-active modes; for example, the peak positions for β-Ga_2O_3 (144, 169, 200, 320, 346, 416, 475, 629, 655 and 766 cm^{-1}) and α-Ga_2O_3 (216, 284, 430, 573, and 689 cm^{-1}) are known [29]. There are no clearly defined modes for δ- and γ-Ga_2O_3 in the literature; mainly only a few broad bands associated with bending and stretching of the Ga-O bond are observed.

Annealing at 300 °C also does not lead to the appearance of new lines, and an increase in temperature to 500 and 700 °C leads to the appearance of weak peaks that are absent in the initial samples. The observed lines in the samples annealed at 500 and 700 °C cannot be unambiguously associated with the known lines from any polytypes. Previously, similar results were observed in [30] and were associated with the formation of nanocrystals of the γ- Ga_2O_3 with low crystallinity. On the other hand, during the formation of Ga_2O_3 nanosized crystalline particles, significant shifts in the positions of the Raman lines [31] and a change in the ratio of their intensities are possible due to different orientations with respect to the polarization of the exciting light.

Increasing the annealing temperature to 900 °C leads to the disappearance of additional peaks, and the spectrum coincides with the spectrum of the initial sample. This may indicate that, at lower temperatures, defective γ-Ga_2O_3 nanocrystals are formed, which either decompose with increasing temperature or transform into another phase that does not contribute to the Raman spectrum, probably due to the low volume concentration of such particles in the layer.

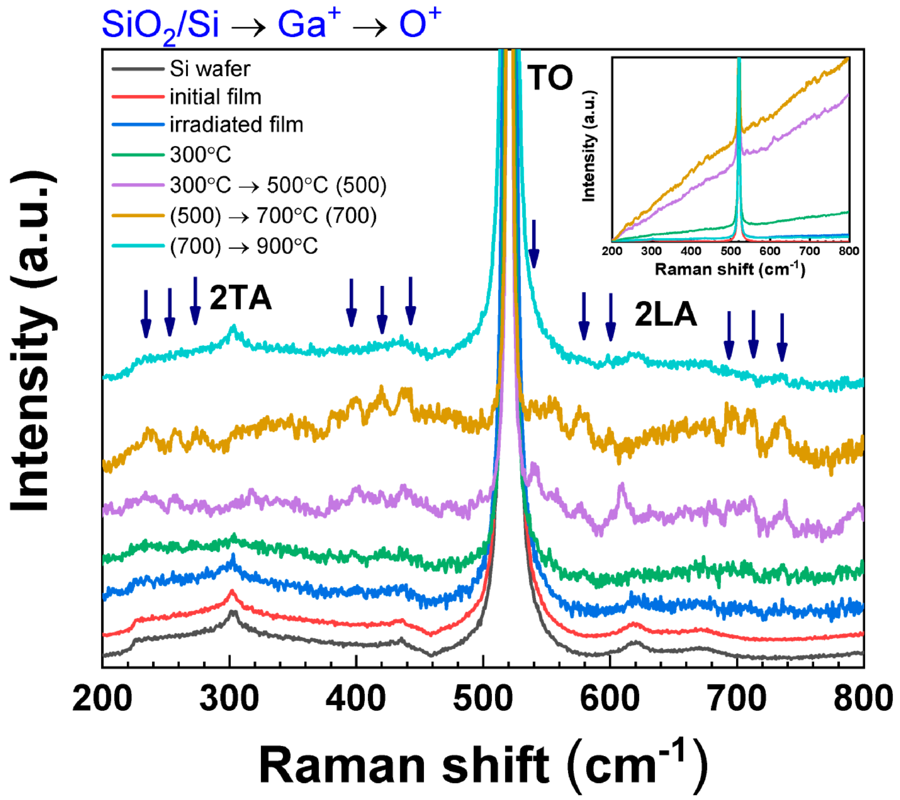

Figure S1. Raman scattering spectra for a SiO_2/Si: (Ga^+ + O^+) sample, before and after sequential annealing. For comparison, the spectra of the initial Si substrate and the initial SiO_2/Si film are also shown. Positions of the unidentified lines are indicated by arrows. Spectra are shown after removing the luminescent background. On the inset, the non-corrected spectra are shown.

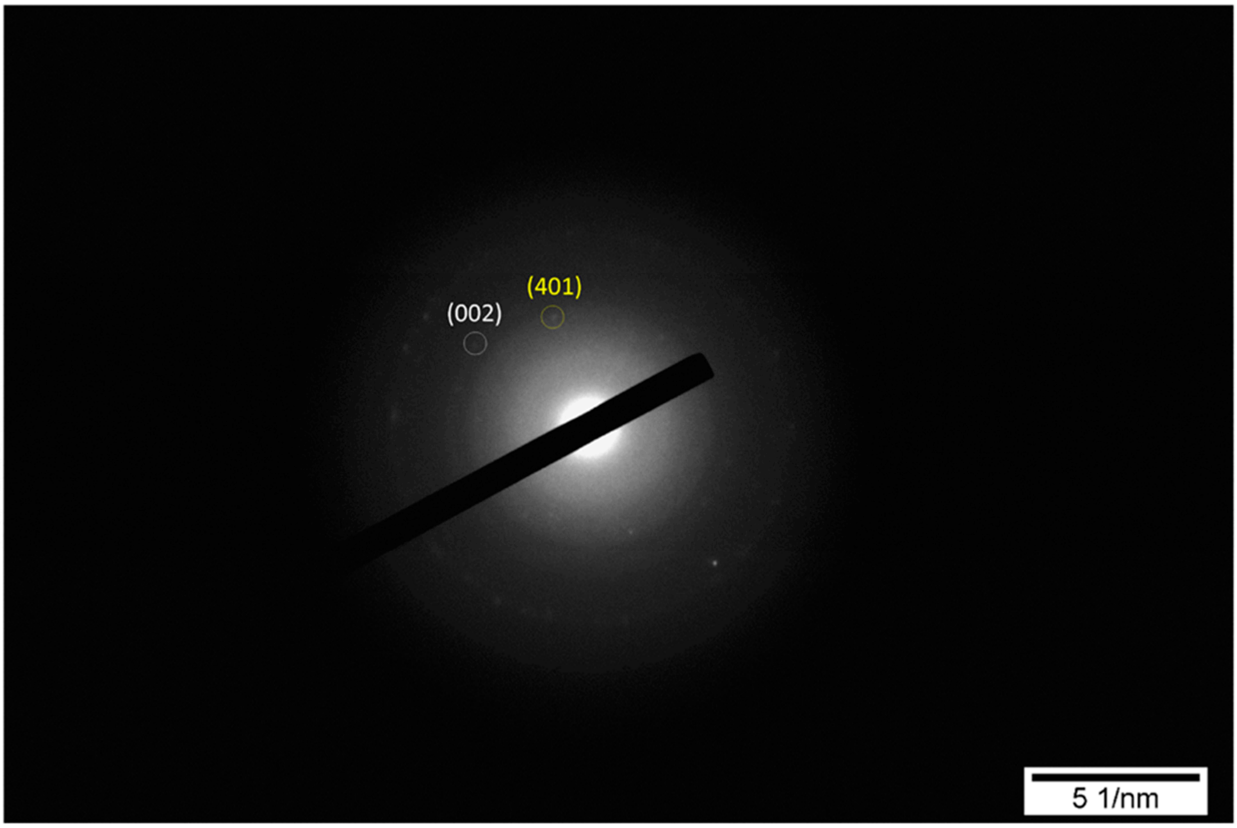

Figure S2. Electron diffraction picture of the SiO_2/Si: (Ga^+ + O^+) structure after final annealing at 900 °C. Spots corresponding to (002) and (401) planes of the nc-Ga_2O_3 (β-phase) are indicated on the picture.

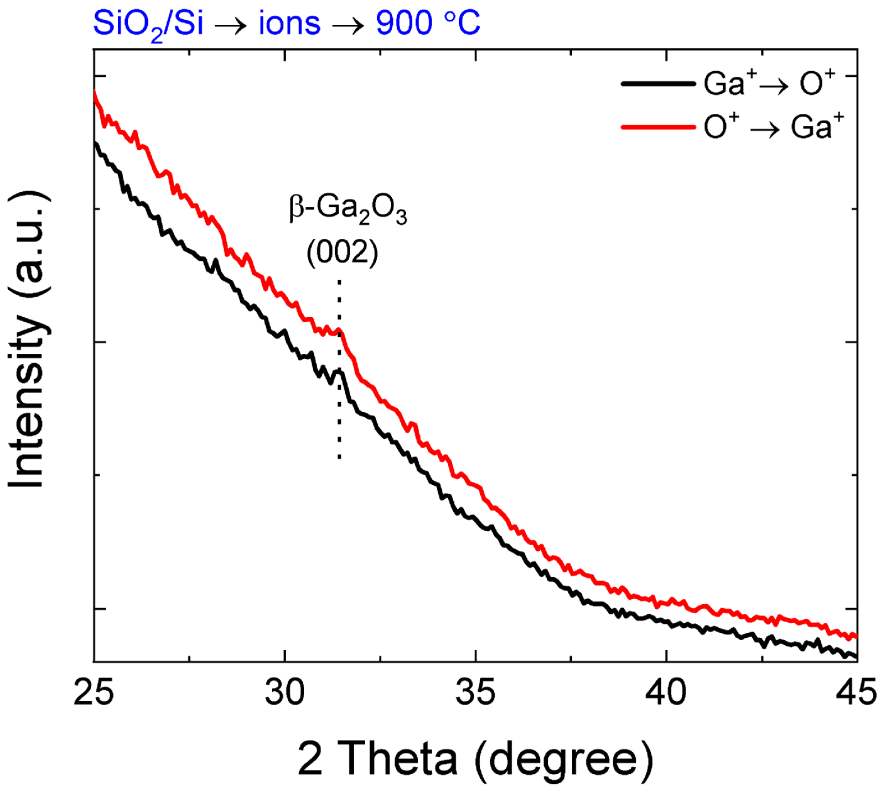

Figure S3. X-ray diffraction spectra of the SiO_2/Si samples implanted with Ga^+ and O^+ ions after final annealing at 900 °C.

References

30. Zhang, X.; Huang, H.; Zhang, Y.; Liu, D.; Tong, N.; Lin, J.; Chen, L.; Zhang, Z.; Wang, X. Phase Transition of Two-Dimensional β-Ga_2O_3 Nanosheets from Ultrathin γ-Ga_2O_3 Nanosheets and Their Photocatalytic Hydrogen Evolution Activities. *ACS Omega* **2018**, *3*, 14469–14476. https://doi.org/10.1021/acsomega.8b01964.
31. Rao, R.; Rao, A.M.; Xu, B.; Dong, J.; Sharma, S.; Sunkara, M.K. Blueshifted Raman Scattering and Its Correlation with the [110] Growth Direction in Gallium Oxide Nanowires. *J. Appl. Phys.* **2005**, *98*, 94312. https://doi.org/10.1063/1.2128044.
